# Physiological Characteristics of Type 1 Diabetes Patients during High Mountain Trekking

**DOI:** 10.1155/2020/8068710

**Published:** 2020-09-18

**Authors:** Bartłomiej Matejko, Andrzej Gawrecki, Marta Wróbel, Jerzy Hohendorff, Teresa Benbenek-Klupa, Dorota Zozulińska-Ziółkiewicz, Maciej T. Malecki, Tomasz Klupa

**Affiliations:** ^1^Department of Metabolic Diseases, Jagiellonian University Medical College, Krakow, Poland; ^2^University Hospital, Krakow, Poland; ^3^Department of Internal Medicine and Diabetology, Poznan University of Medical Sciences, Poznan, Poland; ^4^Department of Internal Medicine, Diabetology and Cardiometabolic Diseases, Silesian Center of Heart Diseases, Zabrze, Poland; ^5^DiabWay, Krakow, Poland

## Abstract

In this study, the aim was to provide observational data from an ascent to the summit of Mount Damavand (5670 meters above sea level (m.a.s.l), Iran) by a group of people with type 1 diabetes (T1DM), with a focus on their physiological characteristics. After a 3-day expedition, 18 T1DM patients, all treated with personal insulin pumps, successfully climbed Mount Damavand. Information was collected on their physiological and dietary behaviors, as well as medical parameters, such as carbohydrate consumption, glucose patterns, insulin dosing, and the number of hypo- and hyperglycemic episodes during this time frame. The participants consumed significantly less carbohydrates on day 3 compared to day 1 (16.4 vs. 23.1 carbohydrate units; *p* = 0.037). Despite this, a gradual rise in the mean daily glucose concentration as measured with a glucometer was observed. Interestingly, the patients did not fully respond to higher insulin delivery as there was no significant difference in mean daily insulin dose during the expedition. There were more hyperglycemic episodes (≥180 mg/dL) per patient on day 3 vs. day 1 (*p* < 0.05) and more severe hyperglycemic episodes (>250 mg/dL) per patient on days 2 (*p* < 0.05) and 3 (*p* < 0.05) vs. day 1. In summary, high mountain trekking is feasible for T1DM patients with good glycemic control and no chronic complications. However, some changes in dietary preferences and an observable rise in glucose levels may occur. This requires an adequate therapeutic response.

## 1. Introduction

The American Diabetes Association recommends that people with type 1 diabetes mellitus (T1DM) should be able to participate in all forms of sports consistent with their desires and goals [1]. As a result of the recent progress in T1DM treatment and monitoring, the number of patients undertaking extreme sports activities has been constantly growing. This includes individuals practicing high mountain trekking. However, research data on their physiological performance as well as health benefits and risks are very limited, and not many clinical recommendations exist in this field [1–8]. This is associated with the fact that very few T1DM patients have been observed at high altitudes for their physiologic, pathophysiologic, and diabetic management, so far. More data on high altitude physiology in patients with diabetes could help provide diabetic-specific guidelines and make a conscious decision about climbing to a high altitude. This could be essential when proposing recommendations concerning diet, insulin therapy, and acclimatization, a process that involves many short- and long-term physiological adaptations occurring to maintain oxygen balance in response to altitude exposure [3, 9]. It is unknown what kinds of changes are necessary in diabetes management strategies during trekking at extreme altitudes (over 5500 m.a.s.l.). In such conditions, increased physical activity may both reduce insulin requirements and cause an altitude-induced rise in counter-regulatory hormone concentration, potentially increasing insulin resistance.

Before trekking, T1DM patients should take into account many other potential problems, such as the effect of delayed absorption of carbohydrate at mealtime, dehydration, high levels of exertion, psychological stress, suppressed appetite, risk of insulin freezing, decreased effectiveness of glucagon owing to possible body glycogen store depletion, unintentional insulin delivery from the insulin pump due to reduced barometric pressure, and many others [1, 3, 5]. One may also hypothesize that existing retinopathy may worsen with the rise of physiological demands for oxygen and its reduced pressure during high-altitude trekking. However, current literature indicates that patients who have no diagnosed complications do not appear to be at a substantial risk for developing new diabetes-related complications [3]. T1DM patients with microvascular complications who wish to undertake travel at altitude should undergo a medical evaluation of conditions that might increase exercise-associated risk; this includes medical history, physical examination, retinal examination, resting/exercise ECG, and/or pulmonary assessments [3]. However, it is of scientific and clinical importance to provide more data in this field [10].

We have previously reported an ascent to Mount Damavand (5670 m.a.s.l.), the highest peak in Iran, by a group of T1DM patients, as well as their performance on insulin pumps during that event [11]. Here, we present further observational data collected during our Damavand expedition, with a focus on the participants' physiological characteristics.

## 2. Materials and Methods

The study was approved by the Local Bioethical Committee. Patients have given their informed consent for the participation in this study.

Our group consisted of individuals with a diverse baseline level of physical activity. There were 4 participants who had completed the “Butchers race” (an 84 km mountain marathon) two months prior to the current expedition, but some others had a baseline physical activity comparable to the general population. This group had some previous experience in high mountain climbing (Alps, 3000 m.a.s.l.) [6]. Basic data related to our 2016 Damavand expedition included the patients' characteristics, glycemic control, and pump functioning, as well as safety issues [11]. In short, Mount Damavand was ascended within 3 days by a group of 19 individuals (17 men, 2 women) all diagnosed with T1DM and treated with a personal insulin pump. They were at a mean age of 32.5 years (range, 23–48 years), had a mean body mass index of 23.8 kg/m^2^ (range, 19.7–30.2 kg/m2), a mean HbA1c level of 6.6% (range, 5.9–7.1%), and a mean diabetes duration of 12.6 years (range, 3–29 years).

All patients were using the blood glucometers that are recommended for their current pump (Accu-Chek Performa Combo (4 patients) (Roche Diagnostics), Contour Link (7), Contour Plus Link 2.4 (7), and Contour Plus (1) (Bayer HealthCare LLC, Diabetes Care). Registration conditions (altitude, temperature, and humidity) for each device used in our study were summarized earlier [6]. The patients were given meals rich in carbohydrates, and no protein or fat meals were recommended. They spent one night at the Polour Resort, Iran (altitude: 2270 m.a.s.l.), after which they drove to Goosfand-Saran (altitude: 3200 m.a.s.l.), where they began their ascent to the shelter (4200 m.a.s.l., 1930 m altitude difference, 6 h of trekking). On day 2, the group climbed to 4700 m.a.s.l. to acclimate to the altitude (3 h) and returned to the shelter (4200 m.a.s.l., 500 m altitude difference, 5 h of trekking). On day 3, 18 T1DM patients decided to challenge the mountain and after 9.5 hours of climbing reached the peak around 11:40 a.m. (1470 m altitude difference, 15 h of trekking).

The following data was collected on the participants' physiological characteristics and dietary behaviors, as well as medical parameters: carbohydrate and fluid consumption, glucose patterns, insulin dosing, number of hypo- and hyperglycemic episodes, blood oxygen saturation, and blood ketone and lactate concentration. A hypoglycemia episode was defined as blood glucose < 70 mg/dL (3.9 mmol/L), while hyperglycemia as blood glucose ≥ 180 mg/dL (10 mmol/L). Additionally, the threshold for severe hyperglycemia was set at 250 mg/dL (13.9 mmol/L). Glucose fluctuations were primarily assessed with blood glucometers. Measurements documenting episodes of hypo- or hyperglycemia within 30-minute periods were counted as one event. All patients were using glucometers recommended for their pumps. They were advised to establish their blood glucose concentrations with the use of glucometers at least 8 times per day: before breakfast and every 1–1.5 hours during breaks and before meals; if needed, additional measurements could be performed. There were 10 (52.5%) T1DM patients using CGM (Medtronic with Enlite sensor 2) and 11 (57.8%) using FGM (FreeStyle Libre technology, Abbott Diabetes Care); among them, 4 were using both systems [11]. As our patients used two different monitoring systems, we decided to assess blood glucose concentrations primarily with glucometers. Baseline glucose data were previously described [11]. In the present paper, we provide further detailed information, comparing the 3 expedition days.

We also estimated acute mountain sickness (AMS) on a scale which evaluated the negative health effects of high altitude, caused by rapid exposure to low amounts of oxygen. Symptoms may include headaches, vomiting, fatigue, trouble with sleeping, and dizziness. Additionally, the Borg scale was used as a method of subjectively rating perceived exertion on the basis of physical signs such as heart rate, breathing rate, and perspiration on a scale of 0–10.

In the paper, we focused on the differences between the 3 days of the expedition using the Borg and AMS scales [10]. We measured lactate blood concentrations at the beginning of the day, i.e., directly before trekking started, and at the highest altitude achieved during the day, up to 5 minutes after reaching the highest point (Lactate Scout, EKF-Diagnostics; registered to work at temperatures ranging from 5°C to 45°C, at 10–85% humidity, and up to an altitude of 4000 m.a.s.l., as per the instruction manual). Ketone blood concentration was also examined at the highest altitude achieved during the day.

To approximate a baseline fitness, a few weeks before the expedition, VO_2_ peak (peak oxygen uptake) was assessed in 13 T1DM patients. We applied a running treadmill (Saturn, HP Germany) test (speed: 6.2 km/h, where at 3-minute intervals, the inclination was increased: 3, 6, 9, 12, 15, 18, 21, and 24°) until the patient was unable to continue due to exhaustion. We used the following devices: a K4b2 analyzer (Cosmed, Italy), a biochemical analyzer Reflotron Plus (Roche, France), a lactate analyzer Biosen S-line (EKF, Germany), and a body composition analyzer (BIA101 Anniversary). VO_2_ peak in mL/kg/min occurring at peak exercise was used to define the subject's aerobic capacity and expressed as the mean value during the last 1 min of the test [12].

No preventive medication to assist acclimatization was used.

Statistically significant differences were set by ANOVA. To make pairwise comparisons between days, we applied the pairwise *t*-test with the Bonferroni correction (*p* < 0.05). Statistical analyses were performed with R 3.6.1 statistical software. The Shapiro-Wilk test served to assess the normality of distribution.

## 3. Results

The observational data collected from all the 19 T1DM participants are summarized in Table 1. During the entire trek, the mean fluid consumption was 2–2.4 L per day, with no significant differences between the expedition days. However, the T1DM patients consumed significantly less carbohydrates on day 3 compared to day 1 (16.4 vs. 23.1 carbohydrate units (1 carbohydrate unit = 10 g of digestible carbohydrates), *p* = 0.037 in post hoc analysis).

**Figure 1:**
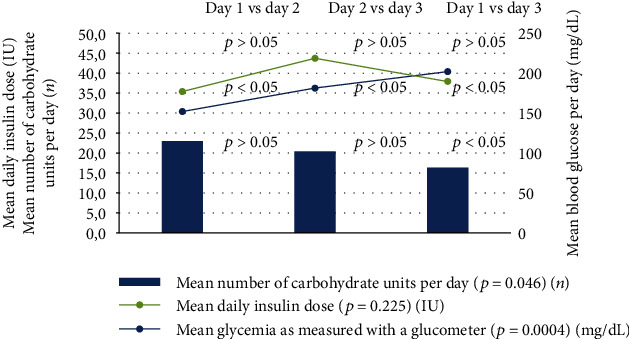
The relationship between mean glycemia, mean carbohydrate unit intake, and daily insulin dose on each expedition day (with *p* values for the comparison between the 3 expedition days).

Despite the drop in carbohydrate consumption (day 3 vs. day 1, *p* < 0.05), a rise in mean daily glucose concentration as measured with a glucometer was observed (Figure 1). Interestingly, the patients did not fully respond to a higher insulin delivery as there was no significant difference in the mean daily insulin dose during the expedition. There were more hyperglycemic episodes per patient on day 3 vs. day 1 (4.8 vs. 2.1, *p* < 0.05) and more severe hyperglycemic episodes per patient on days 2 (1.8, *p* < 0.05) and 3 (1.7, *p* < 0.05) vs. day 1 (0.6) (Table 1). Similar to the glucometer results, the CGM/FGM system data showed significant differences between the expedition days, documenting a gradual increase in blood glucose concentration (Table 1).

The results of the Borg test scale revealed that the perceived exertion was significantly higher at the beginning of days 2 (*p* = 0.0125) and 3 (*p* = 0.0039) vs. day 1. The perceived exertion at the highest altitude achieved during the day was significantly higher on day 3 compared to day 1 (*p* = 0.0134) and day 2 (*p* = 0.0008). There was no correlation between baseline physical fitness and the Borg scale results. AMS symptoms at the beginning of the day were significantly more intense on day 2 (*p* = 0.0001) and day 3 (*p* = 0.0004) vs. day 1.

The blood oxygen saturation measured at the beginning of the day was slightly higher on day 1 than on day 2 (*p* = 0.0017). The blood oxygen saturation measured at the highest altitude reached during the day gradually decreased with the statistically lowest value on day 3 vs. day 1 (*p* ≤ 0.0001) and day 2 (*p* ≤ 0.0001).

**Figure 2:**
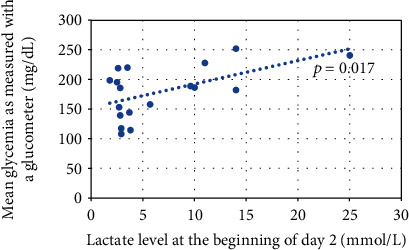
The correlation between lactate concentration at the beginning of day 2 and mean glucometer glycemia on day 2.

There was a numerical increase in mean lactate concentration at the beginning of each of the following days in an absolute value, but no statistical difference was observed. A positive correlation was established between lactate concentration at the beginning of day 2 and mean glucometer glycemia on day 2 (*p* = 0.017, *r* = 0.55; Figure 2). No such correlation was revealed for day 1 or 3 or for lactate concentration at the highest altitude achieved during the day.

The baseline VO_2_ peak equaled 44.9 ± 4.1 mL/kg/min. In accordance with Astrand [13], the VO_2_ peak was classified as very good in 2 cases, as good in 2 cases, as average in 6 cases, as poor in 2 cases, and as very poor in 1 case. There was no correlation between baseline fitness capacity and lactate concentration, Borg scale results, or mean glycemia as measured with a glucometer across the 3 expedition days.

## 4. Discussion

In this observational study, we summarized the physiological data of a group of T1DM patients during a high trekking Damavand expedition. A significant rise in glucose concentration with higher altitudes reached was observed; however, this was not associated with a higher insulin delivery by T1DM patients. Additionally, it was observed that a reduction in carbohydrate consumption occurred on the subsequent expedition days.

Some of our findings are in line with two earlier studies reporting a loss of appetite and higher glucose concentrations at higher altitudes [1, 14]. It has been suggested that transient hyperglycemia occurs during short-term exposure to high altitude, whereas long-term exposure results in lower plasma glucose concentrations [14]. Most studies reported increments in insulin requirement at higher altitudes in patients with T1DM [15–17]. Increased insulin requirements despite exercise were also shown at extreme altitudes [18]. In addition, a positive correlation between AMS symptoms and insulin requirements was observed [14].

The observed diabetes management inertia contributing to hyperglycemia among our patients could be potentially explained by the perceived exertion, which increased together with the altitude reached. It was also postulated that the stress response to high altitude dominates exercise-enhanced insulin sensitivity, resulting in relative hyperglycemia [19]. Of interest, the rise in glucose concentration could not be fully explained by the rise in lactate concentration alone, as the positive correlation was found only for day 2. Thus, some other factors, like AMS and decreased blood oxygen saturation, could have also contributed.

The high baseline level of lactate in our study group requires further comments. One possible explanation is a lack of appropriate acclimatization of our patients and subsequent hypoxia on the tissue level. This shortened acclimatization period occurred due to the delayed arrival to Iran and probably had an impact on the observed results. Some other studies showed that if the exposure to high altitude was sufficiently long, lactate responses to exercise return to those seen at a sea level [20].

On the molecular level, a phenomenon contributing to the increased lactate levels observed at higher altitudes might be increased membrane cotransporters for lactate induced by physical activity [21]. This could cause a reduction in intracellular and an increase in extracellular lactate concentrations. Of note, solely on day 3, we observed that lactate concentrations were higher before climbing than after climbing (as shown in Table 1). There are two possible explanations. First, the reason for this observation might be many missing values of lactate level at the peak of Mount Damavand, which should be considered a shortcoming of this report. The expedition stayed on the peak only for a few minutes, and measurements of lactate were done only for 4 patients. Another explanation could be a “lactate paradox” which defines the phenomenon of a progressive decrease in plasma lactate when increasing altitude during maximum effort [22].

Our study has some obvious limitations, such as a small sample size, gender difference (the majority are men), no CGM data available for all patients, and no detailed information on diet composition. Another issue that should be raised is the relatively short time of the whole ascent and subsequent lack of full acclimatization due to the political situation in Turkey during this investigation (our plane was delayed by one day).

Another important limitation is that all glucose and lactate monitoring devices were used outside of the range of their registration altitude and temperature. Relative humidity might also affect the performance of the devices, but it was not measured in our study. This makes their accuracy and precision uncertain and could have influenced the results. Thus, results obtained in our study must be interpreted with substantial caution. Of note, this shortcoming concerns most T1DM observational studies involving biochemical measurements performed at a very high altitude (1, 2, and 3). Further research is needed to provide a better understanding of the physiology of high-altitude stress markers, such as catecholamines, cortisol, and others.

## 5. Conclusions

In summary, high mountain trekking is feasible for T1DM patients with good glycemic control and no chronic complications. However, some changes in dietary preferences and a subsequent rise in glucose levels may occur. This requires an adequate therapeutic response. Our results also seem to emphasize the necessity of an adequate acclimatization in this group of patients during high mountain expeditions.

## Figures and Tables

**Table 1:** Study group characteristics during the 3-day expedition.

Variable	18 June (3200–4200 m.a.s.l.)				19 June (4200–4700 m.a.s.l.)				20 June (4200–5600 m.a.s.l.)				*p* value^#^
	Mean	SD	Median	Range	Mean	SD	Median	Range	Mean	SD	Median	Range	
Glycemia from FGM (mg/dL)	168	36	164	116–243	219	42	215	155–290	264	45	254	199–338	<0.0001^∗^^~^
Glycemia from CGM (mg/dL)	153	19	146	131–188	163	19	162	137–192	202	30	189	174–261	0.0004^~&^
Glycemia as measured with a glucometer (mg/dL)	153	34	151	98–220	181	40	185	112–251	202	31	208	137–262	0.0004^∗^^~&^
No. of blood glucose measurements per day (glucometer) (*n*)	12.5	4.3	12	5–20	12.4	6.4	12	4–27	14.4	7.6	12	3–27	0.483
Daily insulin dose (IU)	35.6	10.2	33.6	21.1–61.5	43.8	17	45	21.4–94.9	38.1	15.3	35.5	18.2–73.4	0.225
No. of boluses per day (*n*)	7.5	1.9	8	4–10	8.4	2.7	9	5–16	8.5	3.2	8	2–13	0.464
% of basal insulin (%)	39.4	8.6	40	22–63	36.7	9.9	37.2	20–56	40.6	9.4	42	22–56	0.434
Carbohydrate units per day (*n*)	23.1	8.8	20.5	6.5–45	20.4	8.4	18.4	10–42	16.4	5.6	15.2	7.5–29	0.046^~^
Amount of fluids per day (L)	2.4	1	2.1	0.7–4	2	0.8	2	1–4	2.4	0.9	2	1.2–4	0.45
No. of hypoglycemic episodes per patient (*n*)	0.8	1	0.5	0–3	0.3	0.7	0	0–3	0.2	0.7	0	0–3	0.115
No. of hyperglycemic (≥180 mg/dL) episodes per patient (*n*)	2.1	1.1	2.0	0–4	3.3	2.3	3.0	0–9	4.8	3.0	5.5	0–10	0.0025^~^
No. of severe hyperglycemic (>250 mg/dL) episodes per patient (*n*)	0.6	0.7	1	0–2	1.8	1.5	2	0–5	1.7	1.5	1	0–6	0.0165^∗^^~^
AMS scale at the beginning of the day	0.7	0.9	0	0–3	3.4	1.9	4	0–8	3.2	2.2	3	0–7	<0.0001^∗^^~^
AMS scale at the end of the day	4.1	2.4	4	0–8	2.2	1.8	2	0–6	3.3	2.4	3	0–7	0.0669
Borg scale at the beginning of the day	1.1	0.5	1	0–2	2.1	0.9	2	1–4	2.3	1	2	1–4	0.0024^∗^^~^
Borg scale at the highest point of the day	4.4	2.3	4	1–0	4.2	1.8	5	1–6	7	1.9	8	3–9	0.0050^~&^
Lactate concentration at the beginning of the day (mmol/L)	5.7	4.7	4.4	1.3–21	6.7	6	3.6	1.8–25	7.4	7.3	5.1	2.2–25	0.826
Lactate concentration at the highest point of the day (mmol/L)	9.6	7.9	6.8	1.7–25	10.6	6.8	9.6	1.5–25	4.3^!^	2.2	3.6	2–9	0.4062
Blood saturation at the beginning of the day (%)	91.7	5.1	93	73–97	85	6	85	74–93	85.5	14.4	84	64–119	0.0020^∗^
Blood saturation at the highest point of the day (%)	85.2	6.6	85.5	75–97	83	6	80	75–93	78.2	8.1	76	67–95	<0.0001^~&^
Ketone concentration 4200/4700 and 5000 m.a.s.l. (mmol/L)	0.2	0.1	0.2	0.1–0.5	0.2	0.2	0.1	0.1–0.7	0.2	0.2	0.1	0.1–0.6	0.769

1 carbohydrate unit = 10 g of digestible carbohydrates. All variables are means for the entire group. There were 10 patients using CGM and 11 using FGM; among them, 4 individuals used both systems. ^!^Only 4 measurements. ^#^*p* value for the comparison between the 3 expedition days. ^∗^Day 1 statistically significantly different from day 2 (*p* < 0.05). ^~^Day 1 statistically significantly different from day 3 (*p* < 0.05). ^&^Day 2 statistically significantly different from day 3 (*p* < 0.05).

## Data Availability

The data used to support the findings of this study are available from the corresponding author upon reasonable request.
